# Feature optimization in high dimensional chemical space: statistical and data mining solutions

**DOI:** 10.1186/s13104-018-3535-y

**Published:** 2018-07-13

**Authors:** Jinuraj K. R., Rakhila M., Dhanalakshmi M., Sajeev R., Akshata Gad, Jayan K., Muhammed Iqbal P., Andrew Titus Manuel, Abdul Jaleel U. C.

**Affiliations:** 1 Research and Development Centre, Bharathiar University, Marudhamalai Rd, Coimbatore, TamilNadu 641046 India; 2 Department of Chemistry, Malabar Christian College, Calicut, Kerala 673001 India; 3 OSPF-NIAS Drug Discovery Lab, NIAS, Indian Institute of Science Campus, Bengaluru, Karnataka 560012 India; 4 Department of Chemistry, University of Calicut, Malappuram, Kerala 673635 India; 5 Principal Scientist , Cheminformatics, OSPF-NIAS Drug Discovery Lab, NIAS, Indian Institute of Science Campus, Bengaluru, Karnataka 560012 India

**Keywords:** Virtual screening, Z-test, PubChem bioassay, Molecular descriptors, Principal component analysis, Self-organizing maps, Eli Lilly MedChem rules, Molecular similarity

## Abstract

**Objectives:**

The primary goal of this experiment is to prioritize molecular descriptors that control the activity of active molecules that could reduce the dimensionality produced during the virtual screening process. It also aims to: (1) develop a methodology for sampling large datasets and the statistical verification of the sampling process, (2) apply screening filter to detect molecules with polypharmacological or promiscuous activity.

**Results:**

Sampling from large a dataset and its verification were done by applying Z-test. Molecular descriptors were prioritized using principal component analysis (PCA) by eliminating the least influencing ones. The original dimensions were reduced to one-twelfth by the application of PCA. There was a significant improvement in statistical parameter values of virtual screening model which in turn resulted in better screening results. Further improvement of screened results was done by applying Eli Lilly MedChem rules filter that removed molecules with polypharmacological or promiscuous activity. It was also shown that similarities in the activity of compounds were due to the molecular descriptors which were not apparent in prima facie structural studies.

**Electronic supplementary material:**

The online version of this article (10.1186/s13104-018-3535-y) contains supplementary material, which is available to authorized users.

## Introduction

Structure and ligand-based virtual screening methods are widely employed in drug discovery process [1–4]. These methods are high dimensional and complex to analyze which pose some basic challenges. As the virtual screening models built are mathematical, consisting of a large number of dimensions [5] making it difficult to interpret and analyze using ordinary mathematical, statistical or computational methods on computers with lower computational resources. In this study, descriptors were built by molecular descriptor generating and visualization software PowerMV. Few of such software packages are listed in Additional file 1: Table S1.

Molecular descriptors were prioritized by applying principal component analysis (PCA) from the available chemical and biological molecular descriptors. A virtual screening experiment was designed so as to check for any significant effect on the results with and without the application of PCA. For this purpose, training models were built using same PubChem bioassay dataset. PCA provides an efficient way to reduce several independent variables by removing descriptors redundant in a particular study and in turn resulting in a less complex screening model encompassed of a lower number of dimensions.

For this study, AID 1721- a high throughput screened confirmatory bioassay dataset against pyruvate kinase receptor of *Leishmania mexicana* was selected from PubChem. Leishmaniasis is a disease with a wide spectrum of clinical manifestations caused by trypanosomatid protozoan parasites belonging to the genus *Leishmania*. It is one of the major life-threatening tropical diseases globally affecting 12 million people in 98 countries [6]. *Leishmania mexicana* causes both cutaneous and mucocutaneous leishmaniasis. Commonly used FDA approved drugs against *Leishmania mexicana* are sodium stibogluconate, sitamaquine, quinacrine and pentamidine are enlisted in Additional file 2: Table S2. But they were reported to have drug resistance and proven to have severe side effects [7, 8]. So, the chemotherapy based on the above drugs is limited and the need for finding promising druggable molecules with minimal side effects and reasonable ADMET properties gains importance. Under these circumstances, an innovative method such as molecular descriptor based virtual screening was utilized.

Self-organizing maps (SOM) and Eli Lilly MedChem rule filter were applied for further refinement of screening results. SOM is a Kohonen Network [9] based artificial neural network (ANN) is an unsupervised training where networks learn from their own classification of training data without supervision, and higher dimensionality is conserved in the lower dimensional space. It finds a wide range of applications especially in drug repurposing and scaffold hopping [10]. Molecules with polypharmacological/promiscuity activity [11, 12] are another challenge for drug discovery process. Watson et al. developed an algorithm to screen out such compounds from the bioassays [13]. A few of those rules are listed in Additional file 3: Table S3.

## Main text

### Materials and methods

#### Datasets

Publicly available high throughput screened bioassay datasets from PubChem Repository were chosen for the study which was based on NIH Chemical Genomics Center-NCGC assay protocols. The three datasets AID 1721, AID 2559 and AID 2561 used were confirmatory bioassays for *Leishmania mexicana* pyruvate kinase [14] downloaded on September 2013 in SDF format. Pyruvate kinase is responsible for generating ATP from ADP using phosphoenolpyruvate as a substrate [15, 16]. AID 1721 was considered as the training set that had a total of 2,93,196 tested molecules among which 1089 were active and 2,90,104 were inactive. AID 2559 contains 58 actives and 67 inactives. Similarly, AID 2561 contains 37 actives and 148 inactives.

#### Preparation of datasets and molecular descriptor generation

As the inactive set of AID 1721 was a larger one, it was split into 15 subsets using Perl script SplitSDFiles available in MayaChem Tools [17]. The active dataset of AID 1721 was mixed with each of the inactive datasets. Five percent (5%) was randomly selected from each subset resulted in 15 sets each with 1020 molecules were considered as training sets. These 15 sets were considered as training sets. The test set was made by combining active molecules of AID 2559 and AID 2561. A total of 179 descriptors were generated for all the datasets by using PowerMV [18] with 147 pharmacophore fingerprints, 24 weighted burden numbers, and 8 properties. Henceforth, we call these molecular descriptor set as PowD. Calculated properties were transferred into an excel sheet. The bioactivity values were appended as a class attribute, label: class.

#### Sample validation by Z-test

The consistency of the sampling process on AID 1721 was checked by performing Z-test done by XLSTAT **[**19]. Though all the 15 sets passed the Z-test, set-13 was found to have the lowest values for its molecular descriptors and therefore was excluded from further studies.

#### Feature selection and ranking by PCA

PCA was carried out on training datasets to find the most influencing molecular descriptors. This, in turn, could reduce the dimensionality resulting in a less complex virtual screening model. Fourteen weighted burden number descriptors were selected by XLSTAT as principal features that were found contribute most to the principal component F1, and henceforth we designate it as PCAD.

#### Model generation and screening

WEKA 3.6 [20] with a cross-validation of 10 is used for model generation and screening. Models were built with PowD and PCAD for all the training sets. Random forest algorithm was used as it was proven to handle imbalanced datasets [21]. The model performance parameter values such as true positive (TP) rate, true negative (TN) rate, accuracy, kappa, ROC value, F-measure and Matthews correlation coefficient (MCC) etc., associated with both the cases were tabulated.

The test set was screened against all the 14 training models built with PCAD. Panel selection method was implemented to obtain the screening results. Panel selection method is a procedure where a molecule in the test set is considered to be selected if it is selected by 10 or more training sets during the screening process.

#### SOM Analysis

A 5*5 SOM analysis was done for the screened molecules and existing drugs to find the similarity between them. Schrödinger Canvas [22] was used and PCAD as the properties. In this study, SOM with supervised training method was used as supervised learning occurs due to the inclusion of known active FDA approved drugs.

#### Promiscuity analysis

The molecules which showed similarity with the existing FDA approved drugs in SOM analysis were tested by Eli Lilly MedChem rules package to eliminate the molecules showing polypharmacology/promiscuity.

#### In-Silico validation of FDA approved drugs

To find whether the FDA approved drugs pass the same screening criteria, the existing drug molecules were made to undergo screening against the training sets. For this purpose, a dataset was made using FDA approved drugs with PCAD and screened against the training datasets. The panel selection criterion was followed and the results were tabulated.

### Results and discussions

The training and test set used in this study were confirmatory in nature. Since the inactive dataset of AID 1721 was a larger one, it was downsized by splitting into 15 subsets and random selection of 5% from each after mixing active molecules. This procedure enabled to solve the limitations of lower computational resources. A total of 179 molecular descriptors were generated. Z-test was done to check the reliability of the sampling process. The details of Z-test are given in Table 1. Set-13 was eliminated as its values of molecular descriptors were found to be beyond the desirable limits, resulted in 14 training datasets.

**Table 1:** Z-test results for training sets

Sets	WBN ENH 1.00	Z-value	WBN LPH 0.75	Z-value	WBN LPH 1.00	Z-value
Set 1	3.788	0.092	2.790	–	3.652	–
Set 2	3.767	–	2.778	–	3.641	–
Set 3	3.798	0.429	2.811	0.451	3.683	0.776
Set 4	3.781	0.016	2.799	0.010	3.663	0.014
Set 5	3.836	0.999	2.833	0.999	3.704	0.999
Set 6	3.826	0.999	2.832	0.999	3.702	0.999
Set 7	3.839	0.999	2.815	0.731	3.685	0.853
Set 8	3.758	8.259	2.783	–	3.638	–
Set 9	3.785	0.047	2.816	0.789	3.680	0.624
Set 10	3.737	0.475	2.787	0.486	3.642	0.485
Set 11	3.834	0.999	2.842	1.000	3.717	0.999
Set 12	3.854	1.000	2.843	1.000	3.717	0.999
Set 13	3.785	0.494	2.818	0.504	3.681	0.501
Set 14	3.788	0.092	2.821	0.958	3.688	0.933
Set 15	3.817	0.978	2.807	0.194	3.675	0.336
Mean	3.799		2.811		3.678	

#### Wbn enh 1.00, wbn lph 0.75, wbn lph 1.00

Weighted burden number molecular descriptor values. These were continuous descriptors obtained by placing one of the three properties on the diagonal of the Burden connectivity matrix like electronegativity, Gasteiger partial charge or atomic lipophilicity, XLogP. It is common to scale the off-diagonal elements of the connectivity matrix before computing Eigenvalues. The off-diagonal elements were weighted by one of the following values: 2.5, 5.0, 7.5 or 10.0. We use the largest and smallest Eigenvalues. This procedure gives us a total of 24 numerical descriptors. Euclidian distance is used to measure distance instead of Tanimoto distance while calculating continuous descriptors.

PowD was generated for training sets that made input data high dimensional. It was assumed that few subsets of whole molecular descriptors were found to be contributed much towards the activity of the molecules and not the whole set. PCA was applied which enabled in the selection of most relevant molecular descriptors by eliminating the irrelevant ones. The selected molecular descriptors PCAD, which were considered as principal components, are listed in Table 2.

**Table 2:** Weighted Burden number molecular descriptors selected by applying PCA

Serial no.	PCA descriptors
1.	WBN GC H 0.75
2.	WBN GC L 1.00
3.	WBN GC H 1.00
4.	WBN EN L 0.50
5.	WBN EN L 0.75
6.	WBN EN H 0.75
7.	WBN EN L 1.00
8.	WBN EN H 1.00
9.	WBN LP L 0.50
10.	WBN LP H 0.50
11.	WBN LP L 0.75
12.	WBN LP H 0.75
13.	WBN LP L 1.00
14.	WBN LP H 1.00

Statistical models were generated with PowD and PCAD for all the 14 training sets using random forest classifier algorithm. The study used tenfold internal cross-validation to make the model building process ratified, assuring the robustness of the models built. The statistical parameter values of each training set model with PowD and that of PCAD were compared. It was observed that there was a significant hike in statistical parameter values like accuracy, precision, kappa, MCC etc., for the models with PCAD than the models with PowD. The results are shown in Additional file 4: Table S4 and Additional file 5: Table S5. As a result, it was evident that the quality of the virtual screening model was increased to a considerable extent with the application of PCA and this, in turn, should improve the screening results.

As the application of PCA had proven to improve the statistical parameter values, the models built with PCAD were considered for further screening and analysis. The test set was screened against all the 14 training sets. The panel selection method was applied to the selection process and 34 molecules from the test set were selected. Panel selection method applied during the screening process added reliability to the selection process. The results are given in Additional file 6: Table S6.

SOM analysis with 5*5 matrix was done with the screened compounds along with FDA approved drugs to improve the screening results; the output is given in Table 3. “Structurally similar compounds exert similar biological activities” was the concept used by the medicinal chemists to modify biologically active compounds and active principles. However, several structurally similar compounds showed a significantly different mode of action at their target binding site to induce different biological activities [23, 24]. In Table 3, sitamaquine showed similarity with compounds with the PubChem CID 387104 and 828003. The values of it’s of weighted burden number descriptor WBN_GCH_100 were 3.80957, 3.69928 and 3.69448, respectively. The compounds might structurally differ but showed resemblance in activity due to its comparable molecular descriptors values. The molecular descriptor values of compounds listed in Table 3 were given in Additional file 7: Table S10.

**Table 3:**
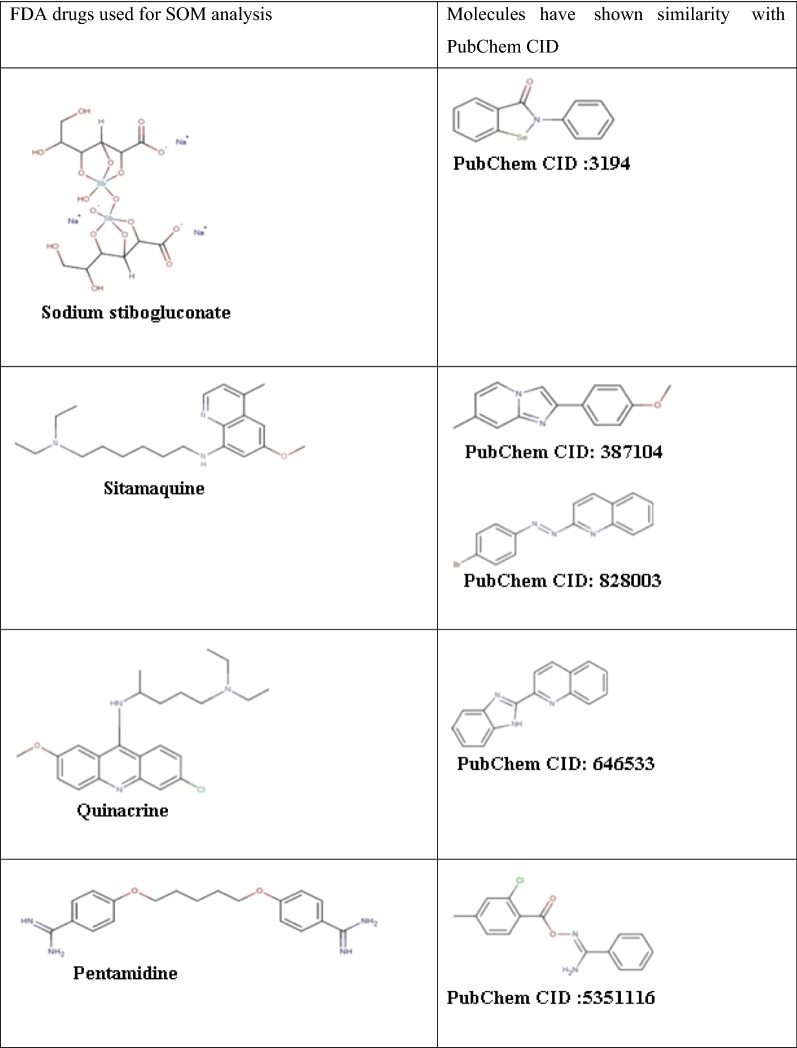
FDA approved drug molecules along with molecules that have shown similarity with drug molecules—SOM (5*5) results

Finally, Eli Lilly MedChem rules were applied to check whether the molecules exhibit any promiscuous action. Two computationally active molecules were screened out by Eli Lilly MedChem analysis that showed the molecules obtained were in agreement with experimentally derived medicinal chemistry rules. The chemical structures of selected compounds are given in Additional file 8: Table S7. The outcome of high throughput screening of bioassays at PubChem was expressed in terms of PubChem activity score. A score of 40–100 denotes active compounds, 1–39 shows inconclusive and a score of 0 denotes inactive compounds. The screened compound with PubChem CID 646533, 3-(1H-1,3-Benzadiol-2-yl)quinoline was 43 while that of 828003, 2-(4-Methoxyphenyl)-7-methylimidazo[1,2-a]pyridine was 83. The dose–response curve quality of the former was a partial curve while that of latter is a complete curve. Both showed an efficacy greater than 80% of control and both the compounds exhibited it’s half maximal efficacy AC_50_, at 91.905 µM concentration, details are listed in Additional file 9: Table S9.

The rationale of using FDA approved drugs for similarity search was justified and validated. The four FDA approved drugs were screened against training sets using PCAD and adopted panel selection method. All the four FDA approved drugs were selected by 10 or more training sets and the results are tabulated in Additional file 10: Table S8. The training and test set used for the study is attached as Additional file 11 and Additional file 12 respectively.

## Conclusion

PCA was able to reduce the number of molecular descriptors which were used during the virtual screening process. This study emphasized that screening carried out with a reduced number of descriptors resulted in increased accuracy. This made it amply clear that PCA successfully removed the redundancy in the input data thereby, improving upon the statistical parameter values as well. It could also be used to extract the characterizing set of molecular descriptors for the activity of molecules against a particular disease or target protein. Using this method along with other methods like Eli Lilly filter and SOM, two molecules were screened out for further processes in the drug discovery and development pipeline. Also, the reason for the similarity in activity among compounds was able to explain in terms of underlying similarities in molecular descriptors.

## Limitations

1. The virtual screening has mainly two methods such as structure-based and ligand-based whereas we emphasized only ligand based method.
2. Among the vast molecular descriptor space, we used only PowerMV molecular descriptors.

## Additional files

**Additional file 1: Table S1.** List of few molecular descriptors generating software packages.

**Additional file 2: Table S2.** Structures of FDA approved drugs against *Leishmania mexicana* selected for the study.

**Additional file 3: Table S3.** Examples of rejection and demerit rules from Eli Lilly MedChem rules.

**Additional file 4: TAble S4.** Statistical parameter values of models with PCAD and PowD for training sets 1 to 7.

**Additional file 5: Table S5.** Statistical parameter values of models with PCAD and PowD for training sets 8 to 15.

**Additional file 6: Table S6.** The screening results of the test set with PCAD against training set. The panel selection scores are also given at the rightmost column.

**Additional file 7: Table S10.** Weighted burden number descriptor values (PCAD) of FDA approved drugs and that of PubChem molecules which were enlisted in Table 3.

**Additional file 8: Table S7.** List of molecules which passed Eli Lilly MedChem filter after SOM analysis.

**Additional file 9: Table S9.** PubChem high throughput screen results of 3-(1H-1,3-Benzadiol-2-yl)quinoline and 2-(4-Methoxyphenyl)-7-methylimidazo[1,2-a]pyridine.

**Additional file 10: Table S8.** Results of Screening of FDA approved drugs against training sets by applying panel selection method.

**Additional file 11.** The 14 training sets used for study which is derived from AID 1721, a high throughput screened, confirmatory bioassay dataset on pyruvate kinase protein target of *Leishmania mexicana*. Training sets are given as ARFF file and have 179 molecular descriptors generated using PowerMV.

**Additional file 12.** The active molecules from AID 2559 and 2561 were considered as the test set. These were high throughput screened confirmatory bioassay dataset. AID 2559 was consisting of 58 active and 67 inactive molecules whereas, AID 2561 was having 37 actives and 148 inactive molecules. The actives from both were combined to get the test set as ARFF file.

## Abbreviations

PCA: principal component analysis
SOM: self-organizing maps
ANN: artificial neural network
PowD: 179 molecular descriptors produced by PowerMV
PCAD: PCA selected molecular descriptors
RF: random forest
TP: true positive
FP: false positive
TN: true negative
FN: false negative
MCC: Matthews correlation coefficient
