# Supplementary material for: Feature optimization in high dimensional chemical space: statistical and data mining solutions
Source: BMC Res Notes. 2018 Jul 13;11:463. doi: 10.1186/s13104-018-3535-y (PMC6044099; doi:10.1186/s13104-018-3535-y)
Supplement: Supplementary file 1 — Additional file 1: Table S1. List of few molecular descriptors generating software packages. [file 13104_2018_3535_MOESM1_ESM.docx]

| **No:** | **Software** | **Type of licensing** | **No.of descriptors** | **Web Source** |
| --- | --- | --- | --- | --- |
| **1.** | E-dragon 1.0 | Free | ~3000 | <http://146.107.217.178/lab/edragon/start.html> |
| **2.** | PowerMV V0.61 | Free(academic version) | 6122 | <https://www.niss.org/research/software/powermv> |
| **3.** | PreADMET | Free-Web version | 2500 | <https://preadmet.bmdrc.kr/> |
| **4.** | MOLGEN 5.0 | Free-Web version | 707 | <http://www.molgen.de/online.html> |
| **5.** | PaDEL | Free | 1875 | <http://www.yapcwsoft.com/dd/padeldescriptor/> |
| **6.** | BlueDesc | Free | 174 | <http://www.ra.cs.uni-tuebingen.de/software/bluedesc/welcome_e.html> |
| **7.** | Dragon 7.0 | Commercial | 5270 | <https://chm.kode-solutions.net/products_dragon.php> |

**Additional Table 1**: List of a few molecular descriptor generating software packages
