# Supplementary material for: Feature optimization in high dimensional chemical space: statistical and data mining solutions
Source: BMC Res Notes. 2018 Jul 13;11:463. doi: 10.1186/s13104-018-3535-y (PMC6044099; doi:10.1186/s13104-018-3535-y)
Supplement: Supplementary file 2 — Additional file 2: Table S2. Structures of FDA approved drugs against Leishmania mexicana selected for the study. [file 13104_2018_3535_MOESM2_ESM.docx]

Additional Table 2: FDA approved drugs against *Leishmania mexicana* selected for the study

| Sodium stibogluconate:  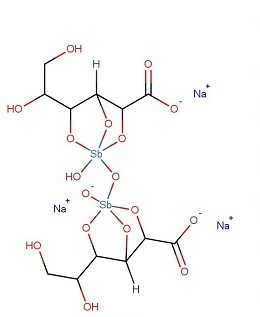 | Sitamaquine  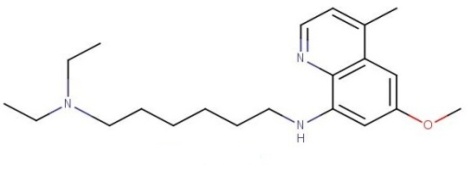 |
| --- | --- |
| Quinacrine  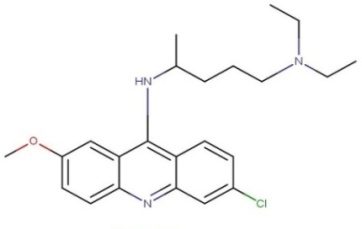 | Pentamidine  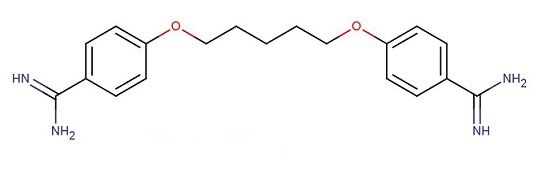 |
