# Supplementary material for: Feature optimization in high dimensional chemical space: statistical and data mining solutions
Source: BMC Res Notes. 2018 Jul 13;11:463. doi: 10.1186/s13104-018-3535-y (PMC6044099; doi:10.1186/s13104-018-3535-y)
Supplement: Supplementary file 3 — Additional file 3: Table S3. Examples of rejection and demerit rules from Eli Lilly MedChem rules. [file 13104_2018_3535_MOESM3_ESM.docx]

**Additional Table 3**: Examples of demerit and rejection rules contained in Eli Lilly MedChem rules.

| **No:** | **Rule** | **Rule class** | **Sub class** | **Demerits** |
| --- | --- | --- | --- | --- |
| **1.** | activated_ester | acylating | acyl-QQ | 160 |
| **2.** | acyl_sulphonamide | acylating | diacyl | 30 |
| **3.** | schiff_base | aldehyde | schiff | 160 |
| **4.** | alkyl_iodide | alkylating | halide | 160 |
| **5.** | 8_hydroxyquinoline | chelator | misc | 160 |
| **6.** | bromine | halogen | count | 34 |
| **7.** | too_many_atoms | misc | count | 7 |
| **8.** | abnormal_valance | misc | valance | 160 |
| **9.** | nitro | nitrogen | nitro | 60 |
| **10.** | hydrazine_cyclic | nitrogen | N-N | 20 |
| **11.** | caumarin | nuisance | misc | 50 |
| **12.** | phthalimide | protecting group | count | 50 |
| **13.** | peroxide | redox | misc | 160 |
| **14.** | no_rings | ring | count | 30 |
| **15.** | quinine_para | vinyl | michael | 160 |
