# Supplementary material for: Feature optimization in high dimensional chemical space: statistical and data mining solutions
Source: BMC Res Notes. 2018 Jul 13;11:463. doi: 10.1186/s13104-018-3535-y (PMC6044099; doi:10.1186/s13104-018-3535-y)
Supplement: Supplementary file 4 — Additional file 4: TAble S4. Statistical parameter values of models with PCAD and PowD for training sets 1 to 7. [file 13104_2018_3535_MOESM4_ESM.docx]

Additional Table 4:Statistical parameter values of models with PCAD and PowD for training sets 1 to 7

| Statistical Parameters | Set-1(179) | Set-1 (14) | Set-2 (179) | Set-2 (14) | Set-3 (179) | Set-3 (14) | Set-4 (179) | Set-4 (14) | Set-5 (179) | Set-5 (14) | Set-6 (179) | Set-6 (14) | Set-7 (179) | Set-7 (14) |
| --- | --- | --- | --- | --- | --- | --- | --- | --- | --- | --- | --- | --- | --- | --- |
| Accuracy | 97.35 | 99.8 | 97.84 | 99.51 | 96.47 | 98.53 | 96.961 | 99.02 | 97.45 | 98.82 | 96.961 | 99.12 | 96.76 | 98.73 |
| Kappa | 0.4396 | 0.972 | 0.4138 | 0.9066 | 0.138 | 0.755 | 0.303 | 0.843 | 0.309 | 0.764 | 0.158 | 0.848 | 0.3176 | 0.805 |
| TN rate | 100 | 100 | 100 | 100 | 100 | 100 | 100 | 100 | 100 | 100 | 100 | 99.9 | 100 | 100 |
| TP rate | 28.9 | 94.7 | 26.7 | 83.3 | 7.7 | 61.5 | 18.4 | 73.7 | 18.8 | 62.5 | 8.8 | 76.5 | 19.5 | 68.3 |
| FN rate | 71.1 | 5.3 | 73.3 | 16.7 | 92.3 | 38.5 | 81.6 | 26.3 | 81.3 | 37.5 | 91.2 | 23.5 | 80.5 | 31.7 |
| FP rate | 0 | 0 | 0 | 0 | 0 | 0 | 0 | 0 | 0 | 0 | 0 | 0.1 | 0 | 0 |
| Precision(for negative class) | 97.3 | 99.8 | 97.8 | 99.5 | 96.5 | 98.5 | 96.9 | 99 | 97.4 | 98.8 | 97 | 99.2 | 96.7 | 98.7 |
| Precision (for positive class ) | 100 | 100 | 100 | 100 | 100 | 100 | 100 | 100 | 100 | 100 | 100 | 96.3 | 100 | 100 |
| Recall (for negative class) | 100 | 100 | 100 | 100 | 100 | 100 | 100 | 100 | 100 | 100 | 100 | 99.9 | 100 | 100 |
| Recall (for positive class) | 28.9 | 94.7 | 26.7 | 83.3 | 7.7 | 61.5 | 18.4 | 73.7 | 18.8 | 62.5 | 8.8 | 76.5 | 19.5 | 68.3 |
| F- measure (for negative class) | 98.6 | 99.7 | 98.9 | 99.7 | 98.2 | 99.2 | 98.4 | 99.5 | 98.7 | 99.4 | 98.5 | 99.5 | 98.3 | 99.3 |
| F-measure(for positive class) | 44.9 | 97.3 | 42.1 | 90.9 | 14.3 | 76.2 | 31.1 | 84.8 | 31.6 | 76.9 | 16.2 | 85.2 | 32.7 | 81.2 |
| ROC | 0.988 | 1 | 0.944 | 1 | 0.903 | 0.94 | 0.893 | 0.915 | 0.976 | 0.997 | 0.95 | 0.999 | 0.899 | 0.985 |
| MCC | 0.53 | 0.97 | 0.51 | 0.91 | 0.27 | 0.78 | 0.42 | 0.85 | 0.43 | 0.79 | 0.29 | 0.85 | 0.43 | 0.82 |
| Negative predictive value | 28.95 | 99.8 | 97.83 | 99.5 | 96.46 | 98.49 | 96.94 | 98.99 | 97.44 | 98.8 | 96.95 | 99.19 | 96.74 | 98.69 |
| F1 Score | 44.7 | 97.3 | 42.11 | 90.91 | 14.29 | 76.19 | 31.11 | 84.85 | 31.58 | 76.92 | 16.22 | 65.82 | 32.65 | 81.16 |

Set-1(179) denotes dataset 1 with 179 molecular descriptors - PowD,Set-1(14) denotes dataset 1 with 14 molecular descriptors selected by PCA, PCAD.TN rate – True Negative rate, TP rate- True Positive rate , FN rate-False Negative rate,FP rate- False negative rate , ROC-Receiver Operating Characteristic curve , MCC- Matthews Correlation Coefficient.
