# Supplementary material for: Feature optimization in high dimensional chemical space: statistical and data mining solutions
Source: BMC Res Notes. 2018 Jul 13;11:463. doi: 10.1186/s13104-018-3535-y (PMC6044099; doi:10.1186/s13104-018-3535-y)
Supplement: Supplementary file 5 — Additional file 5: Table S5. Statistical parameter values of models with PCAD and PowD for training sets 8 to 15. [file 13104_2018_3535_MOESM5_ESM.docx]

Additional Table 5:Statistical parameter values of models with PCAD and PowD for training sets 8 to 15

| Statistical Parameters | Set-8 (179) | Set-8 (14) | Set-9 (179) | Set-9 (14) | Set-10 (179) | Set-10 (14) | Set-11 (179) | Set-11 (14) | Set-12 (179) | Set-12 (14) | Set-14 (179) | Set-14 (14) | Set-15 (179) | Set-15 (14) |
| --- | --- | --- | --- | --- | --- | --- | --- | --- | --- | --- | --- | --- | --- | --- |
| Accuracy | 96.86 | 98.43 | 96.373 | 98.824 | 100 | 98.824 | 97.25 | 98.824 | 97.156 | 99.02 | 96.177 | 98.431 | 97.65 | 99.412 |
| Kappa | 0.153 | 0.6964 | 0.1348 | 0.818 | 4.26 | 0.836 | 0.325 | 0.787 | 0.1165 | 0.7952 | 0.047 | 0.7424 | 0.3267 | 0.8859 |
| TN rate | 100 | 100 | 100 | 100 | 100 | 100 | 100 | 100 | 99.9 | 100 | 100 | 100 | 100 | 100 |
| TP rate | 8.6 | 54.3 | 7.5 | 70 | 2.3 | 72.7 | 20 | 65.7 | 6.7 | 66.7 | 2.5 | 60 | 20 | 80 |
| FN rate | 91.4 | 45.7 | 92.5 | 30 | 97.7 | 27.3 | 80 | 34.3 | 93.3 | 33.3 | 97.5 | 40 | 80 | 20 |
| FP rate | 0 | 0 | 0 | 0 | 0 | 0 | 0 | 0 | 0.1 | 0 | 0 | 0 | 0 | 0 |
| Precision(for negative class) | 96.9 | 98.4 | 96.4 | 98.8 | 95.8 | 98.8 | 97.2 | 98.8 | 97.2 | 99 | 96.2 | 98.4 | 97.6 | 99.4 |
| Precision (for positive class ) | 100 | 100 | 100 | 100 | 100 | 100 | 100 | 100 | 66.7 | 100 | 100 | 100 | 100 | 100 |
| Recall (for negative class) | 100 | 100 | 100 | 100 | 100 | 100 | 100 | 100 | 99.9 | 100 | 100 | 100 | 100 | 100 |
| Recall (for positive class) | 8.6 | 54.3 | 7.5 | 70 | 2.3 | 72.7 | 20 | 65.7 | 6.7 | 66.7 | 2.5 | 60 | 20 | 80 |
| F- measure (for negative class) | 98.4 | 99.2 | 98.1 | 99.4 | 97.8 | 99.4 | 98.6 | 99.4 | 98.6 | 99.5 | 98 | 99.2 | 98.8 | 99.7 |
| F-measure(for positive class) | 15.8 | 70.4 | 14 | 82.4 | 4.4 | 84.2 | 33.3 | 79.3 | 12.1 | 80 | 4.9 | 75 | 33.3 | 88.9 |
| ROC | 0.863 | 0.923 | 0.879 | 0.933 | 0.9 | 0.971 | 0.848 | 0.917 | 0.938 | 1 | 0.843 | 0.926 | 0.9 | 0.965 |
| MCC | 0.29 | 0.73 | 0.27 | 0.83 | 0.15 | 0.85 | 0.44 | 0.81 | 0.2 | 0.81 | 0.16 | 0.77 | 0.44 | 0.89 |
| Negative predictive value | 96.85 | 98.36 | 96.36 | 98.79 | 95.78 | 98.79 | 97.24 | 98.8 | 97.25 | 99 | 96.17 | 98.39 | 97.64 | 99.4 |
| F1 Score | 15.79 | 70.37 | 13.95 | 82.35 | 4.44 | 95.65 | 33.33 | 79.31 | 12.12 | 80 | 4.88 | 75 | 33.33 | 88.89 |

Set**–**8(179) denotes dataset 8 with 179 molecular descriptors, PowD. Set-8(14) denotes dataset 8 with 14 molecular descriptors selected by PCA, PCAD. TN rate – True Negative rate, TP rate- True Positive rate , FN rate-False Negative rate,FP rate- False negative rate , ROC-Receiver Operating Characteristic curve , MCC- Matthews Correlation.
