# Supplementary material for: Feature optimization in high dimensional chemical space: statistical and data mining solutions
Source: BMC Res Notes. 2018 Jul 13;11:463. doi: 10.1186/s13104-018-3535-y (PMC6044099; doi:10.1186/s13104-018-3535-y)
Supplement: Supplementary file 8 — Additional file 8: Table S7. List of molecules which passed Eli Lilly MedChem filter after SOM analysis. [file 13104_2018_3535_MOESM8_ESM.docx]

Additional Table 7:Molecules which passed Eli Lilly MedChem filter after SOM analysis

| 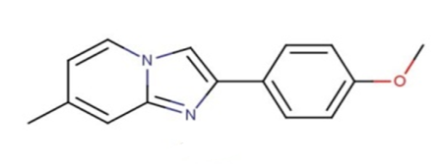  **2-(4-Methoxyphenyl)-7-methylimidazo[1,2-a]pyridine** | 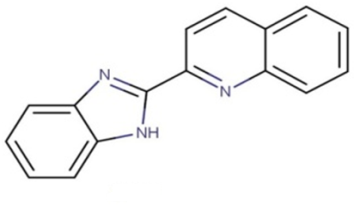  **3-(1H-1,3-Benzadiazol-2-yl)quinoline** |
| --- | --- |
