# Supplementary material for: Feature optimization in high dimensional chemical space: statistical and data mining solutions
Source: BMC Res Notes. 2018 Jul 13;11:463. doi: 10.1186/s13104-018-3535-y (PMC6044099; doi:10.1186/s13104-018-3535-y)
Supplement: Supplementary file 10 — Additional file 10: Table S8. Results of Screening of FDA approved drugs against training sets by applying panel selection method. [file 13104_2018_3535_MOESM10_ESM.docx]

| **Molecule name** | Set-1 | Set-2 | Set-3 | Set-4 | Set-5 | Set-6 | Set-7 | Set-8 | Set-9 | Set-10 | Set-11 | Set-12 | Set-14 | Set-15 |
| --- | --- | --- | --- | --- | --- | --- | --- | --- | --- | --- | --- | --- | --- | --- |
| Sodium stibogluconate | **🗸** | **🗸** | **🗸** | **🗴** | **🗸** | **🗸** | **🗸** | **🗸** | **🗸** | **🗴** | **🗸** | **🗸** | **🗴** | **🗴** |
| Sitamaquine | **🗸** | **🗸** | **🗸** | **🗸** | **🗸** | **🗸** | **🗸** | **🗸** | **🗸** | **🗴** | **🗸** | **🗸** | **🗴** | **🗴** |
| Quinacrine | **🗸** | **🗸** | **🗸** | **🗸** | **🗴** | **🗸** | **🗸** | **🗴** | **🗸** | **🗸** | **🗸** | **🗸** | **🗴** | **🗴** |
| Pentamidine | **🗸** | **🗸** | **🗸** | **🗸** | **🗸** | **🗸** | **🗸** | **🗸** | **🗸** | **🗴** | **🗸** | **🗸** | **🗴** | **🗴** |

**Additional Table 8**: Screening of FDA approved drugs against training sets by applying panel selection method.
